# Evaluating small extracellular vesicle-based vaccination across heterologous *Salmonella* strains isolated from wastewater

**DOI:** 10.1128/iai.00485-24

**Published:** 2025-01-13

**Authors:** Lisa E. Emerson, Saloni Bhimani, Andrew L. Rainey, Anthony T. Maurelli, Mariola J. Ferraro

**Affiliations:** 1 Microbiology and Cell Science Department, IFAS, University of Florida 209721 , Gainesville, Florida, USA; 2 Clinical and Translational Science Institute, University of Florida 535747 , Gainesville, Florida, USA; 3 Emerging Pathogens Institute, University of Florida 145775 , Gainesville, Florida, USA; 4 Department of Environmental and Global Health, University of Florida 271714 , Gainesville, Florida, USA; University of Pennsylvania, Philadelphia, Pennsylvania, USA

**Keywords:** *Salmonella*, wastewater-based epidemiology (WBE), small extracellular vesicles, vaccine, antigenic proteins, immune response, proteomics, non-typhoidal *Salmonella*

## Abstract

*Salmonella* infections pose significant public health challenges worldwide. The diversity of *Salmonella* strains, particularly those isolated from environmental and clinical sources, necessitates innovative approaches to prevention and treatment. Previous research has shown that small extracellular vesicles (sEVs) produced by macrophages during *Salmonella* Typhimurium infection can induce robust immune responses when used as a vaccine, offering complete protection in systemic infection models. In this study, we isolated 120 *Salmonella* strains from qPCR *invA*-positive wastewater samples collected in Gainesville, FL. These strains underwent enrichment, selection, and biochemical confirmation, followed by serotyping and whole genome sequencing. Two isolates, *Salmonella enterica* subsp. diarizonae (Diarizonae) and *S. enterica* serovar Enteritidis, were selected for further analysis based on community prevalence and clinical severity. We also assessed the ability of sEVs produced by *S*. Typhimurium-infected macrophages to induce immune responses against these heterologous and circulating strains in mice. Immunization with sEVs induced robust antigen-specific SIgA and IgG responses against *S*. Typhimurium, Enteritidis, and Diarizonae, with high titers observed in sera and fecal samples. Proteomic analysis revealed differential expression of proteins in these strains, including antigenic proteins present in sEVs such as OmpA, FliC, or OmpD. Moreover, this study highlights the role of wastewater-based epidemiology (WBE) as a tool for environmental surveillance, offering a complementary perspective on *Salmonella* dynamics within a population. Integrating WBE with traditional surveillance methods, along with the promising results of sEV-based vaccination, provides a pragmatic strategy for developing effective preventative measures against *Salmonella* infections, addressing the diversity of non-typhoidal *Salmonella* strains.

## INTRODUCTION

*Salmonella,* a genus of gram-negative bacteria, imposes a substantial global health burden, accounting for over 95 million cases of salmonellosis annually (1). These bacteria primarily inhabit the intestinal tracts of humans and animals, spreading through the shedding of live bacteria in stool (2). The transmission of *Salmonella* occurs predominantly via the ingestion of contaminated food or water, but any contact with feces or *Salmonella*-contaminated fomites poses a risk (2). Although over 2,600 serovars of *Salmonella* exist, several cause typhoid fever, whereas all others cause Non-Typhoidal *Salmonella* (NTS) infections (3). Although most infections are self-limiting, severe cases can progress to invasive Non-Typhoidal *Salmonella* (iNTS) infection, potentially becoming lethal without proper treatment (4). Although all serovars can cause iNTS infection, several serovars cause the heaviest burden of iNTS: Dublin, Choleraesuis, and Typhimurium (5). Despite extensive research, an effective prevention strategy against NTS remains elusive. Recent work in our lab has introduced a promising preventative approach using macrophage-derived small extracellular vesicles (sEVs) to induce both cellular and humoral immunity against NTS (6). Although macrophages are fundamental components of the host’s innate immune defense against bacterial infections, they play a paradoxical role in *Salmonella* infection. The pathogen invades and exploits these phagocytic cells for survival and replication, effectively using them as a Trojan horse to spread within the host (7–10). However, our research has shown that infected macrophages can produce extracellular vesicles that act as “messages in a bubble” for other cells, thereby contributing to both innate and adaptive immunities against this bacterium (6, 11–13). We have also demonstrated that these vesicles can completely protect infected mice against the lethal effects of systemic infection when delivered as a cell-free vaccine (6). To move from mechanistic research to translational studies, the next step involves assessing the efficacy of this preventative strategy against community-derived and heterogeneous strains to enhance the translational relevance of our work. Thus, it is important to determine whether immunity generated by sEVs using a laboratory strain can generate immune responses against strains circulating within a community.

When designing vaccines against *Salmonella*, it is crucial to consider the genetic and antigenic diversities present in natural populations, beyond the laboratory strains (14). Laboratory strains lack the variability observed in environmental and clinical isolates, which can significantly impact vaccine effectiveness. To identify conserved antigens, it is necessary to analyze diverse genome sequences to ensure broad protection. Rare mutations in natural strains can affect critical immune determinants that laboratory strains might not reveal (15). The predominant focus on laboratory strains of *S*. serovar Typhimurium, such as UK-1, SL1344, and NCTC 12023, in current research, poses significant limitations. These commonly used laboratory strains fail to adequately represent the diversity of NTS and have not been associated with human disease. Specifically, the UK-1 strain originated from a horse, whereas the SL1344 and NCTC 12023 strains were initially isolated from cattle (16). Although NTS infections can occur across diverse environmental niches, the pursuit of a potential human vaccine necessitates the use of strains specifically associated with humans. Collaborating with existing surveillance networks offers the opportunity to test new therapies on circulating human NTS strains, thereby enhancing the translational impact of research findings. Here, wastewater-based epidemiology (WBE) stands as a robust environmental surveillance tool, adept at monitoring pathogen circulation, delineating disease trends, and identifying potential outbreaks within a community (17). This method involves the analysis of biomarkers of disease present in influent wastewater, which encompasses the collective input (urine and feces) from all community residents connected to a municipal sewer system (18). These biomarkers include molecular chemical markers, antibiotic resistance genes, nucleic acids of infectious pathogens, and viable pathogens discerned through culture-based methods (18). Notably, NTS is shed through the feces of infected individuals, allowing for the culture of viable NTS from wastewater samples for subsequent isolation and identification (19, 20). In contrast to the current standard method of NTS disease surveillance, which relies on clinical surveillance of treated individuals (19), WBE offers a unique advantage and has recently gained attention, particularly during and after the COVID-19 pandemic (18, 20–25). Clinical surveillance excels in providing accurate information at the individual level but falls short in capturing subclinical cases within the population—those cases that do not seek care. Various factors, including asymptomatic infections, limited healthcare access, and personal choices influenced by low disease severity, geographical, or economic barriers, contribute to the underrepresentation of subclinical cases in clinical surveillance. WBE, however, overcomes this gap by sampling wastewater from the entire population, creating a pooled sample that effectively fills the voids left by clinical surveillance. Proposed as a population-level disease surveillance tool, WBE complements the data generated through clinical surveillance. It indiscriminately collects clinical and subclinical NTS, providing a more comprehensive and inclusive perspective on NTS dynamics within a population (18).

In this study, we isolated and sequenced circulating *Salmonella* strains from the wastewater of Gainesville, FL, selecting two isolates that represent clinical and subclinical cases. We assessed the effectiveness of an sEV vaccine against these strains, determining that animals vaccinated with an sEV vaccine generated using *S*. Typhimurium produce antibodies that recognize these circulating strains, Enteritidis and Diarizonae. Comparative analysis with the laboratory strain *S. enterica* serovar Typhimurium x3761 UK-1 provided insights into the potential of sEV-derived vaccines against real-world NTS strains. This integrated approach, combining sEV-derived vaccine research with surveillance and testing using WBE, aims to overcome barriers to translating NTS vaccine research into practice.

## RESULTS

### Isolation of *Salmonella* strains from wastewater

**Fig 1:**
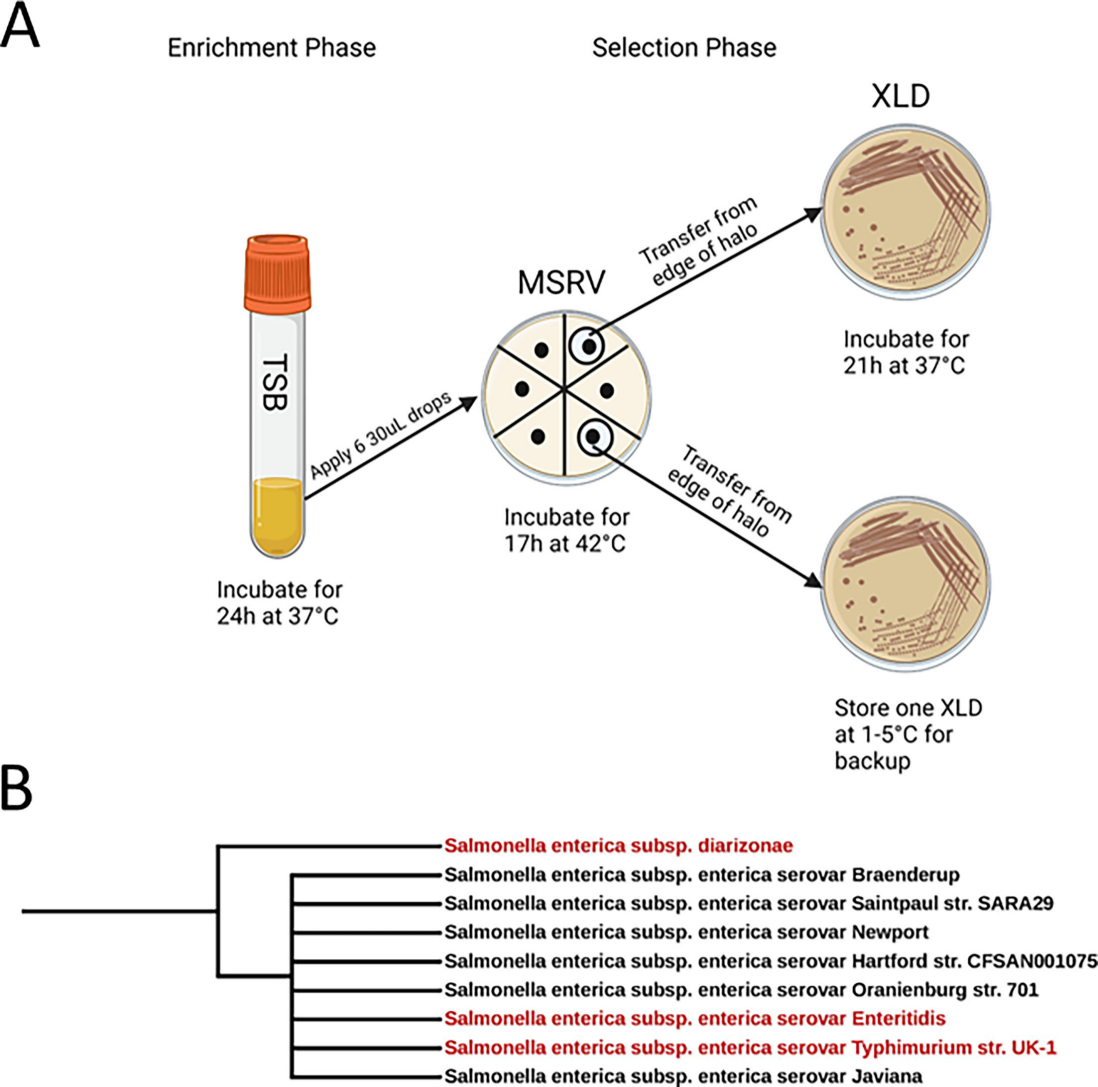
Isolation and characterization of *Salmonella* strains from wastewater samples. (**A**) A total of 120 *Salmonella* isolates were collected from qPCR-*invA*-positive wastewater samples in Gainesville. The isolation process involved an initial enrichment phase in TSB, followed by selection on MSRV plates and further biochemical confirmation on XLD plates. Serotyping and sequencing were performed by the State of Florida Department of Health using SeqSero2, with successful serotyping of 21 isolates. (**B**) Phylogenetic analysis indicating the taxonomy and evolutionary distance between the selected *Salmonella* isolates.

We attempted bacterial isolation from 79 *invA* RT-qPCR-positive raw wastewater samples and recovered 120 NTS isolates from 15 (19%) of those samples. The isolation process involved an initial enrichment phase where the samples were grown in tryptic soy broth (TSB), followed by the selection on modified semi-solid rappaport-Vassiliadis (MSRV) plates and further selection and biochemical confirmation on Xylose Lysine Deoxycholate (XLD) plates (Fig. 1A). These strains underwent sequencing by the State of Florida Department of Health. These isolates were subsequently sequenced by the Florida Department of Health. Serotypes were identified for 21 isolates using the k-mer-based SeqSero 2 algorithm (26), and the results for these serotypes are shown in Table 1. The antimicrobial resistance (AMR) genes for each isolate were determined using the AMRFinderPlus tool . The 21 serotyped isolates represent a diverse range of serotypes, including Braenderup, Diarizonae, Enteritidis, Hartford, Javiana, Newport, Nima, Oranienburg, Saintpaul, Thompson, and Typhimurium. The SeqSero analysis revealed consistent antigenic profiles for each serotype, with notable commonalities such as the 7,h,n,z15 profile for Braenderup and 9,m:- for Enteritidis.

**TABLE 1:** *Salmonella* isolates from wastewater

WGS ID	Biosample ID	SRA ID	SeqSero Serotype	SeqSero AgProfile	AMR genotype
PNUSAS333745	SAMN33391517	SRS16844982	Braenderup	7:e,h:e,n,z15	mdsA, mdsB
PNUSAS290213	SAMN30237449	SRR21003488	Braenderup	7:e,h:e,n,z15	mdsA, mdsB
PNUSAS303872	SAMN33368690	SRS16824028	Braenderup	7:e,h:e,n,z15	mdsA, mdsB
PNUSAS303876	SAMN33368694	SRS16824735	Braenderup	7:e,h:e,n,z15	mdsA, mdsB
PNUSAS290248	SAMN30237433	SRR21003472	Diarizonae	61:c:1,5,7	
PNUSAS333751	SAMN33391522	SRS16844978	Enteritidis	9:g,m:-	mdsA, mdsB
PNUSAS303896	SAMN33368678	SRS16824026	Enteritidis	I 9:g,m:1,5	mdsA, mdsB
PNUSAS328384	SAMN32811050	SRS16514795	Enteritidis	9:g,m:-	mdsA, mdsB
PNUSAS333750	SAMN33391528	SRS16844994	Hartford	7:y:e,n,x	mdsA, mdsB
PNUSAS333758	SAMN33391518	SRS16844985	Javiana	9:l,z28:1,5	mdsA, mdsB
PNUSAS290206	SAMN30237458	SRR21003498	Newport	8:e,h:1,2	mdsA, mdsB
PNUSAS290219	SAMN30237446	SRR21003495	Newport	8:e,h:1,2	mdsA, mdsB
PNUSAS333740	SAMN33391533	SRS16844980	Newport	8:e,h:1,2	mdsA, mdsB
PNUSAS303868	SAMN33368675	SRS16824012	Nima	28:y:1,5	mdsA, mdsB
PNUSAS303869	SAMN33368676	SRS16824020	Oranienburg	I 7:m,t:-	mdsA, mdsB
PNUSAS333753	SAMN33391523	SRS16844995	Saintpaul	4:e,h:1,2	mdsA, mdsB
PNUSAS333739	SAMN33391521	SRS16845025	Saintpaul	4:e,h:1,2	mdsA, mdsB
PNUSAS303887	SAMN33368697	SRS16824005	Saintpaul	4:e,h:1,2	mdsA, mdsB
PNUSAS303877	SAMN33368692	SRS16824025	Thompson	7:k:1,5	mdsA, mdsB
PNUSAS303886	SAMN33368695	SRS16824729	Typhimurium	I 4,[5],12:i:-	mdsA, mdsB
PNUSAS303897	SAMN33368677	SRS16824022	Typhimurium	4:i:1,2	aph(3'')-Ib, aph(6)-Id,mdsA, mdsB, sul2

Two wastewater isolates, *S. enterica* serovar Diarizonae (61:c:1,5,7; Table 1) and *S. enterica* serovar Enteritidis (9:g,m:-; Table 1), were selected for further analysis based on community prevalence and clinical severity. Serovar Enteritidis causes the most significant burden of illness nationwide, and Diarizonae is associated with more severe clinical outcomes. The taxonomy of the selected isolates indicates an evolutionary distance between strains (Fig. 1B). Subsp. Diarizonae is a different subspecies than both serovar Enteritidis and serovar Typhimurium, which both belong to the subspecies enterica.

### Differential invasion of laboratory and wastewater-derived *Salmonella* strains

**Fig 2:**
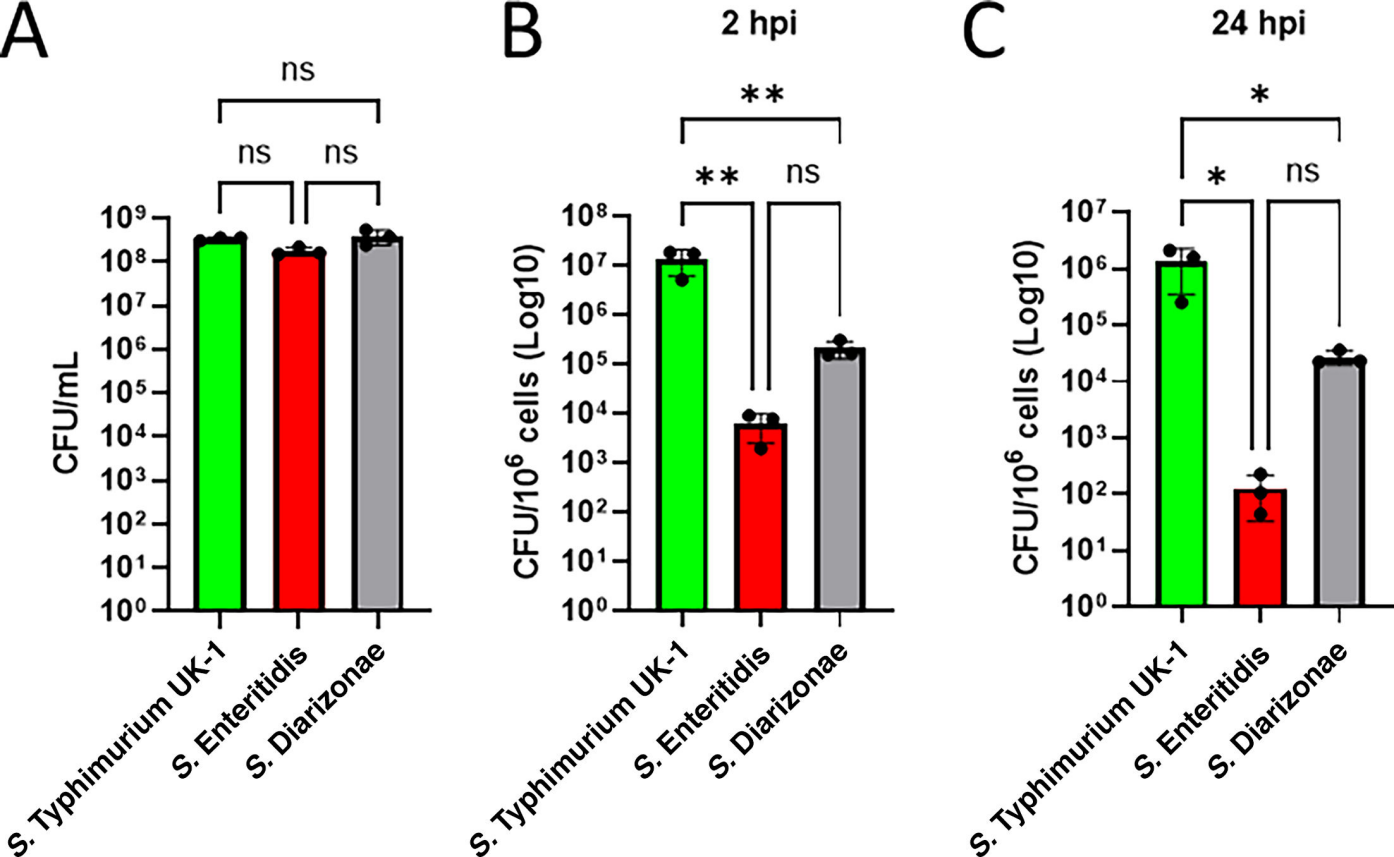
Differential invasion of laboratory and wastewater-derived *Salmonella* strains. (**A**). Viability assessment of the laboratory strain *S*. Typhimurium UK-1 and two wastewater-derived strains (*S*. Enteritidis and *S*. Diarizonae) at OD_600_ = 0.5. Statistical significance was determined using one-way ANOVA with Tukey’s multiple comparisons test. (**B-C**). RAW 264.7 cells were infected with *Salmonella* strains at a multiplicity of infection (MOI) of 30:1, followed by a gentamicin protection assay. Intracellular bacterial counts (CFU per 1 million cells) were measured at 2 hpi (**B**) and 24 hpi (**C**) for *S*. Typhimurium UK-1, *S*. Enteritidis, and *S*. Diarizonae. Statistical significance was determined using one-way ANOVA, with significance levels indicated by asterisks: *P < 0.05, **P < 0.01, and ***P < 0.001; ns indicates not significant. Error bars represent the standard error of the mean (SEM).

The gentamicin protection assay was used to compare the invasion capabilities of a laboratory strain of *Salmonella* Typhimurium (UK-1) and two wastewater-derived strains, *S*. Diarizonae (61:c:1,5,7; Table 1) and *S*. Enteritidis (9:g,m:-; Table 1). First, there was no difference in the viability of these strains at OD_600_ = 0.5 (Fig. 2A), which we used for infection and tracking of intracellular survival studies. The laboratory strain, *S*. Typhimurium UK-1, demonstrated the highest invasion efficiency at both time points analyzed. At 2 hours post-infection (hpi), *S*. Typhimurium UK-1 exhibited significantly higher intracellular colony forming units (CFUs) per 1 million cells compared with both *S.* Enteritidis and *S.* Diarizonae, which had approximately 50% fewer intracellular bacteria (Fig. 2B). This trend persisted at 24 hpi, with *S.* Typhimurium UK-1 continuing to show the highest CFU per 1 million cells (Fig. 2C). These results indicate that *S*. Typhimurium UK-1 has superior invasion efficiency and potentially greater intracellular survival compared with the wastewater-derived serovars, *S*. Enteritidis and *S*. Diarizonae.

### Testing the antibody responses to sEV vaccination across heterologous *Salmonella* strains in mice

Previous research from our laboratory has demonstrated that sEVs produced by macrophages during *Salmonella* Typhimurium infection (Fig. S1), when administered intranasally to BALB/c mice, induce robust antigen-specific SIgA and IgG responses as well as T cell-mediated responses against *S*. Typhimurium (6, 11). These immune responses provide complete protection in a systemic infection model of *Salmonella* infection (6). Here, *S*. Typhimurium-infected RAW264.7 macrophages were used as the source of sEVs, which were subsequently administered to BALB/c mice.

**Fig 3:**
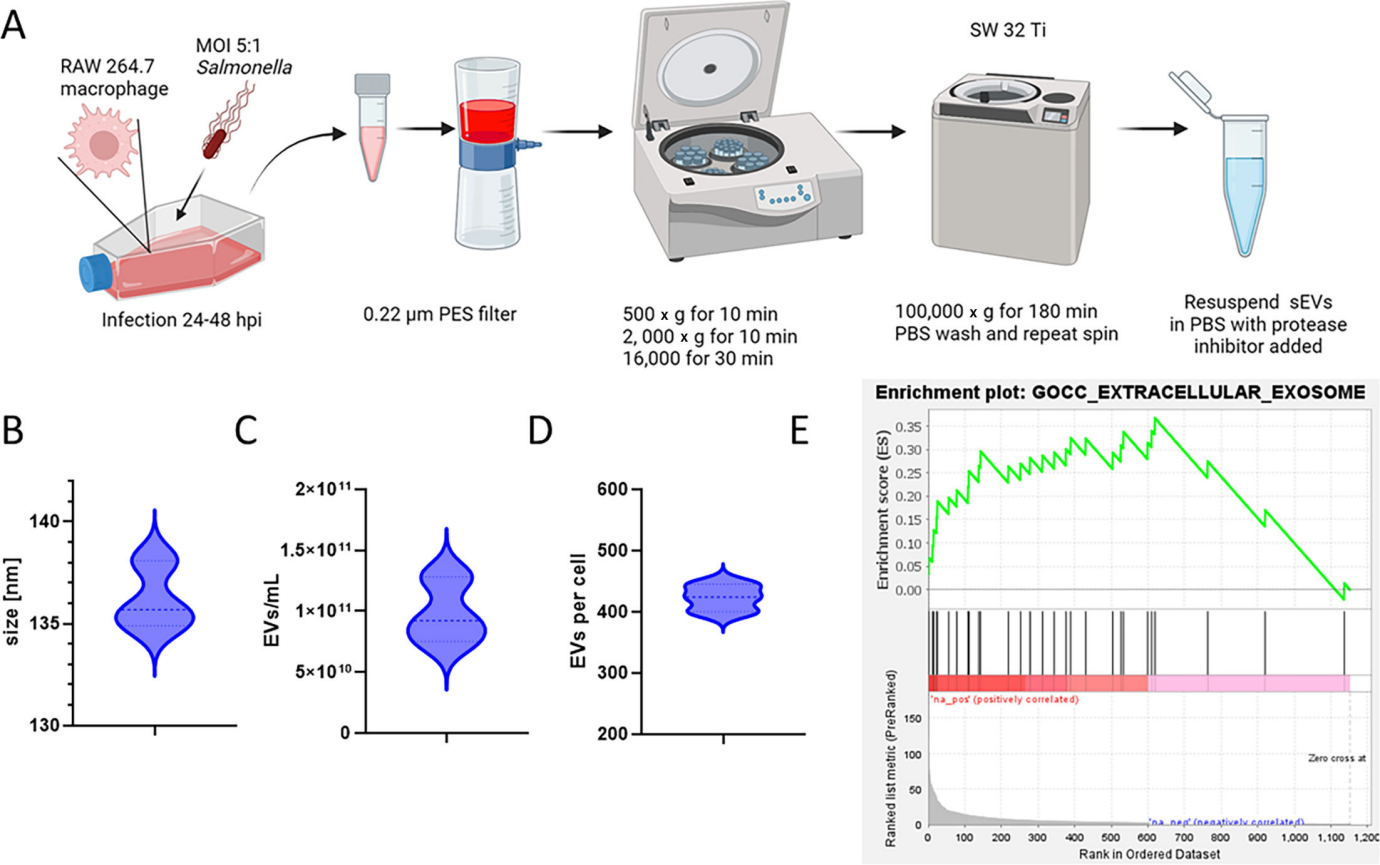
Isolation and characterization of sEVs from *Salmonella*-infected RAW 264.7 macrophages. (**A**) Schematic representation of the sEV isolation process. RAW 264.7 macrophages infected with *Salmonella* Typhimurium (MOI 5:1) were harvested at 24 and 48 hpi. Supernatants were filtered (0.22 µm PES) and subjected to sequential centrifugation steps (500 × *g*, 2,000 × *g*, and 16,000 × *g*) to remove debris, followed by ultracentrifugation at 100,000 × *g* with a PBS wash. The final sEV pellet was resuspended in PBS with protease inhibitors, and size/concentration was measured using NanoSight and ZetaView instruments. (**B**) Violin plot showing the particle size distribution of isolated sEVs, with the shape representing the distribution frequency of measured sizes as determined by ZetaView. (**C**) Violin plot depicting the concentration of sEVs, reflecting the abundance of isolated vesicles as measured by ZetaView. (**D**) Violin plot illustrating the ratio of particle number to macrophage (cell) count, indicating the yield of sEVs per cell. (**E**) Enrichment plot for GO cellular compartment (CC) terms, highlighting “extracellular exosome” as one of the top significant terms, indicating the presence of exosomal markers in the isolated sEV sample. GSEA was used to calculate the enrichment score (ES), with the x-axis representing the rank in ordered data and the y-axis showing ES. Peaks suggest a high concentration of proteins related to extracellular exosomes.

The isolated sEVs had an average size of 136.2 nm (Fig. 3A) and a concentration of 9.86 × 10^10^ particles/mL, as determined by nanoparticle tracking analysis (NTA) using ZetaView (Fig. 3B). On average, each macrophage produced 423 EVs (Fig. 3C). Gene set enrichment analysis (GSEA) of the sEV proteome revealed significant enrichment of the gene ontology (GO) cellular component (CC) category associated with the “extracellular exosome” gene set in the top-ranked portion of the data set (Fig. 3C). The enrichment score peaked at 0.35, indicating a strong positive correlation between this gene set and the high-ranking genes (*Actg1, Hspa8, Sdcbp, Pdcd6ip, Anxa2, Anxa1, Anpep, Aldoa, Tfrc, Gars1, Cd9, Clic1, Ybx1, Ahnak, Nsun2, Tsg101, Rab5b, Cd47, Hspa4, Rab11a, Ist1, Lamp2, Icam1, Sri, Cd81*, and *Rap2b*). Most of the genes within this set were clustered in the top ranks of exosomal proteins, suggesting a crucial role for extracellular exosomes in modulating immune responses in vaccinated subjects.

**Fig 4:**
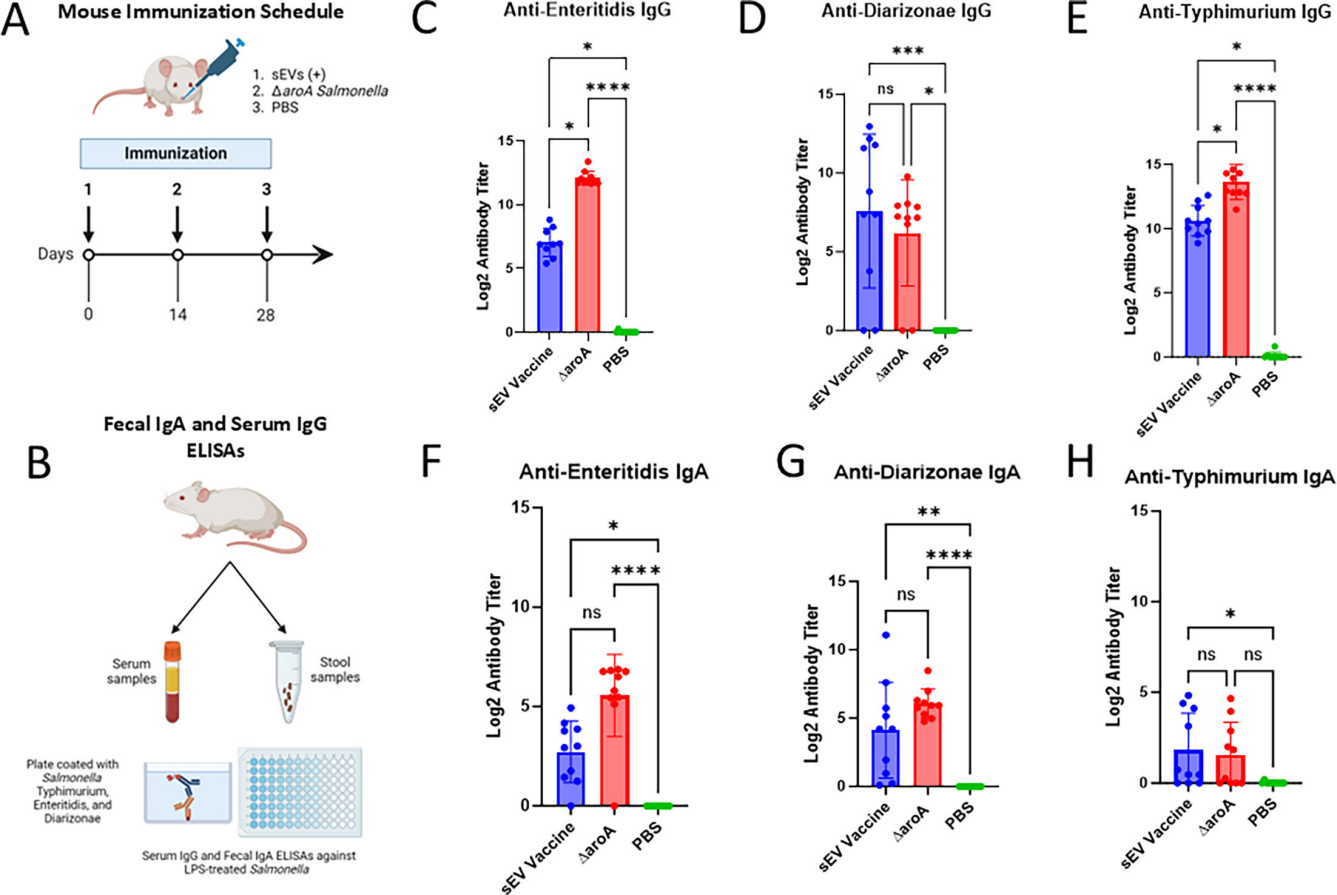
IgG and IgA antibody responses to sEV vaccination across heterologous *Salmonella* strains in mice. (**A**) Experimental design for testing the antibody responses to sEV vaccination. *S.* Typhimurium-infected RAW264.7 macrophages were used to generate sEVs, which were administered intranasally to BALB/c mice in three doses. The ∆*aroA Salmonella* strain served as a positive vaccination control, administered orally, whereas PBS was delivered intranasally as a negative control. (**B**) Schematic representation of the experimental setup for assessing SIgA and IgG responses against heterologous *Salmonella* strains isolated from wastewater, including serovars Enteritidis and Diarizonae compared with Typhimurium. (**C-E**) Serum IgG titers against *S*. Enteritidis (**C**), *S*. Diarizonae (**D**), and *S*. Typhimurium (**E**) were measured by ELISA. (**F-H**) Fecal SIgA titers against *S*. Enteritidis (**F**), *S*. Diarizonae (**G**), and S. Typhimurium (**H**) measured by ELISA. A Kruskal-Wallis test was used to determine significant differences in medians across the three groups. Statistical significance is denoted by asterisks, with *P < 0.05, **P < 0.01, and ***P < 0.001.

These characterized sEVs were then administered to BALB/c mice intranasally in three doses (Fig. 4A). We assessed the ability of these sEVs to induce anti-*Salmonella* SIgA antibodies against heterologous *Salmonella* strains isolated from wastewater, specifically serovars Enteritidis and Diarizonae (Fig. 4B). The ∆*aroA Salmonella* strain served as a positive vaccination control, administered orally as an auxotrophic vaccine strain, whereas phosphate-buffered saline (PBS) was delivered intranasally as a negative control. Fecal samples were collected from individual mice and examined for the presence of SIgA anti-*Salmonella* endpoint titers, with enzyme-linked immunosorbent assays (ELISAs) performed against three strains: the *S.* Typhimurium strain used to generate the sEV vaccine, Enteritidis, and Diarizonae.

Interestingly, serum IgG titers showed statistically significant differences between animals receiving PBS and those vaccinated with sEVs derived *in vitro* from *S*. Typhimurium UK-1-infected macrophages, particularly for Enteritidis and Diarizonae serovars (Fig. 4C through E). However, for the Enteritidis and Typhimurium strains, the live attenuated Δ*aroA S*. Typhimurium-vaccinated group exhibited significantly higher IgG titers compared with the sEV-vaccinated groups (Fig. 4C and E), whereas this was not observed for Diarizonae (Fig. 4D). For SIgA responses, sEV administration resulted in elevated titers of anti-Enteritidis (Fig. 4F), anti-Diarizonae (Fig. 4G), and anti-Typhimurium (Fig. 4H) antibodies. As expected, immunization with the live attenuated ΔaroA *S*. Typhimurium strain (positive control) induced strong IgG and SIgA responses against all tested serovars, although the SIgA response to Typhimurium was not statistically significant. In contrast, PBS administration (negative control) did not elicit antibody response across any serovars (Fig. 4C through H).

Overall, the ELISA results indicate that vaccination with sEVs derived from *S*. Typhimurium lab strain-infected macrophages effectively elicits antibody responses in BALB/c mice against additional heterologous Salmonella serovars, specifically Enteritidis and Diarizonae.

### Proteomic characterization of *Salmonella* wastewater-derived strains compared with a lab strain

**Fig 5:**
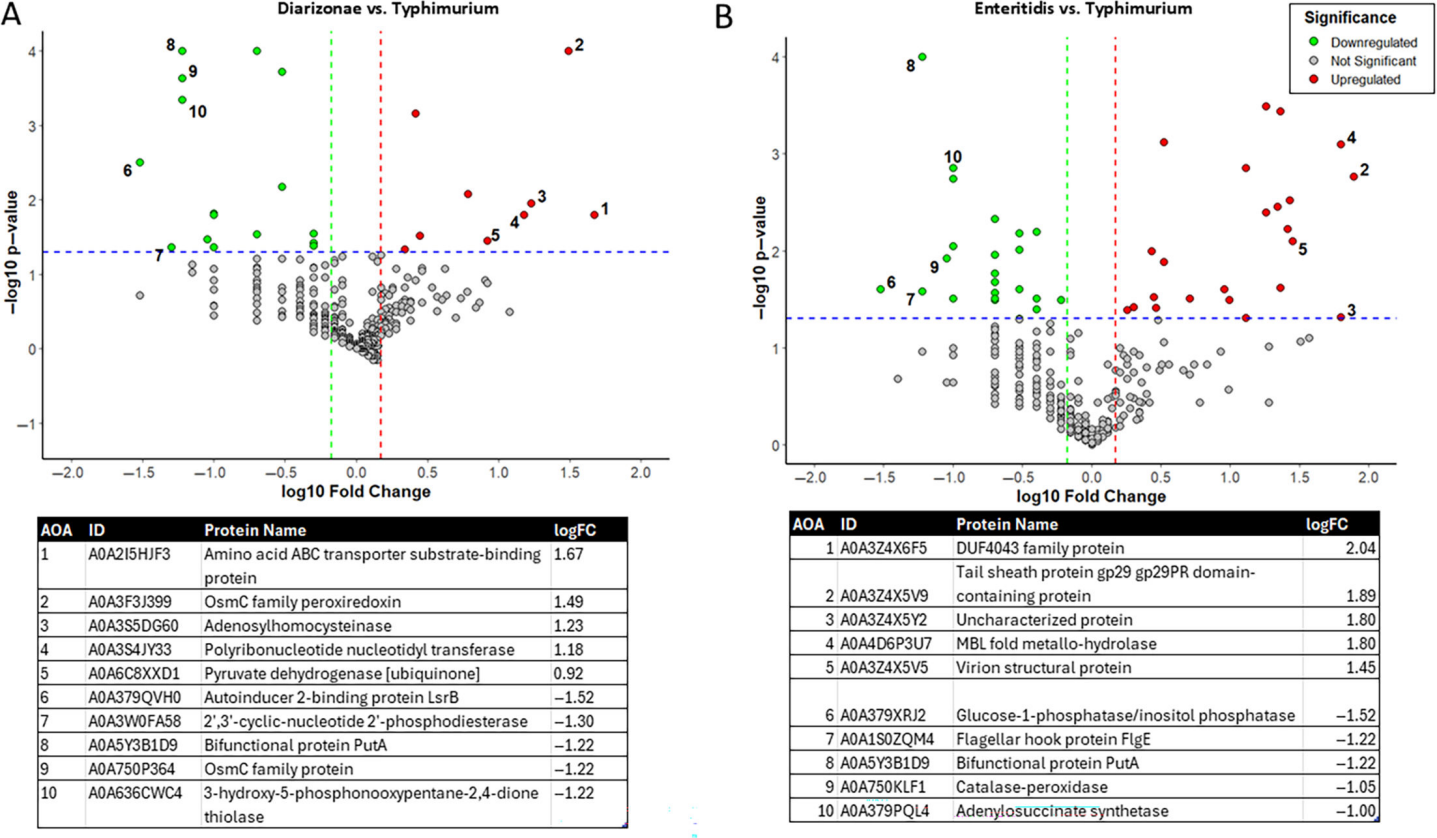
Comparisons of proteomes of *S.* Diarizonae, *S.* Enteritidis, and *S.* Typhimurium strains. **(A**) Volcano plot showing differential protein abundance in *S*. Diarizonae compared with *S*. Typhimurium antigen preparation. (**B**) Volcano plot showing differential protein abundance in *S*. Enteritidis compared with *S*. Typhimurium antigen preparations. Proteins are color-coded by significance and regulation direction: green for downregulated proteins, red for upregulated proteins, and gray for non-significant changes. The x-axis represents the log10 fold change, and the y-axis represents −log10 *P*-value, with dashed lines marking significance thresholds. Tables detailing labeled proteins are provided below each graph.

To further understand the antigenic differences and potentially link them to the observed antibody responses, we analyzed the proteomes of three tested *Salmonella* strains grown to mid-log phase, the same phase used for ELISA assays. Our objective was to identify proteins that could serve as potential antigens, as identified in our prior study (11), and determine if these proteins exhibit altered expression levels between the serovars under identical growth conditions. We used label-free quantification via Orbitrap Fusion mass spectrometry, followed by GO term analysis, which revealed significant changes in the expression of several proteins among the strains (Fig. 5; Table S1).

The proteomic comparison between wastewater-derived *S*. Diarizonae (61:c:1,5,7; Table 1) and laboratory strain *S*. Typhimurium UK-1 revealed significant differences in protein expression. In *S*. Diarizonae, several proteins were upregulated, including OsmC family peroxiredoxins (A0A3F3J399, A0A750P364) and molecular chaperone OsmY (A0A5X6ECX6), indicating enhanced stress responses. Other upregulated proteins, such as polysaccharide deacetylase (A0A1S0ZXM1) and adenosylhomocysteinase (A0A3S5DG60), suggest increased catabolic and metabolic activities. Conversely, downregulated proteins in *S*. Diarizonae include alcohol dehydrogenase (A0A3U2RRP0) and large ribosomal subunit protein uL18 (A0A3Y4SQS0), indicating reduced NAD+ activity and ribosomal function. Additionally, the downregulation of proteins like glucarate dehydratase (A0A0H2WSQ7) and malate synthase (A0A3Z0VAZ2) suggests diminished catabolic and oxidoreductase activities. In summary, *S*. Diarizonae showed an upregulation of stress response and metabolic proteins, whereas proteins related to ribosomal function and specific catabolic processes were downregulated compared to *S*. Typhimurium.

Similarly, the comparative proteomic analysis of wastewater-derived *S*. Enteritidis (9:g,m:-; Table 1) and laboratory strain *S*. Typhimurium UK-1 revealed several differences. In *S*. Enteritidis, numerous proteins were upregulated, including virion structural proteins (A0A3Z4X6D8, A0A3Z4X 5V5, A0A5HJF3), phage tail protein (A0A3Z4X6F6), and peptidase Do (A0A2X4WT57), indicating increased virion-associated components and proteolytic activities. Additionally, fumarate hydratase class II (A0A748A3K1) and long-chain fatty acid transport protein (A0A0D6FAL8) suggest altered metabolic pathways. Conversely, downregulated proteins in *S*. Enteritidis include adenylosuccinate synthetase (A0A379PQL4), glycerol kinase (A0A760G2Q1), and glucarate dehydratase (A0A0H2WSQ7) involved in biosynthetic and metabolic processes. The downregulation of flagellin (Q6V2G5) and catalase-peroxidase (A0A750KLF1) suggests a reduction in proteins controlling motility and oxidative stress response. In summary, *S*. Enteritidis exhibited an upregulation of virion structure, metabolism, and fatty acid transport proteins, whereas downregulated proteins were linked to metabolic processes, motility, and oxidative stress response compared with *S*. Typhimurium.

**Fig 6:**
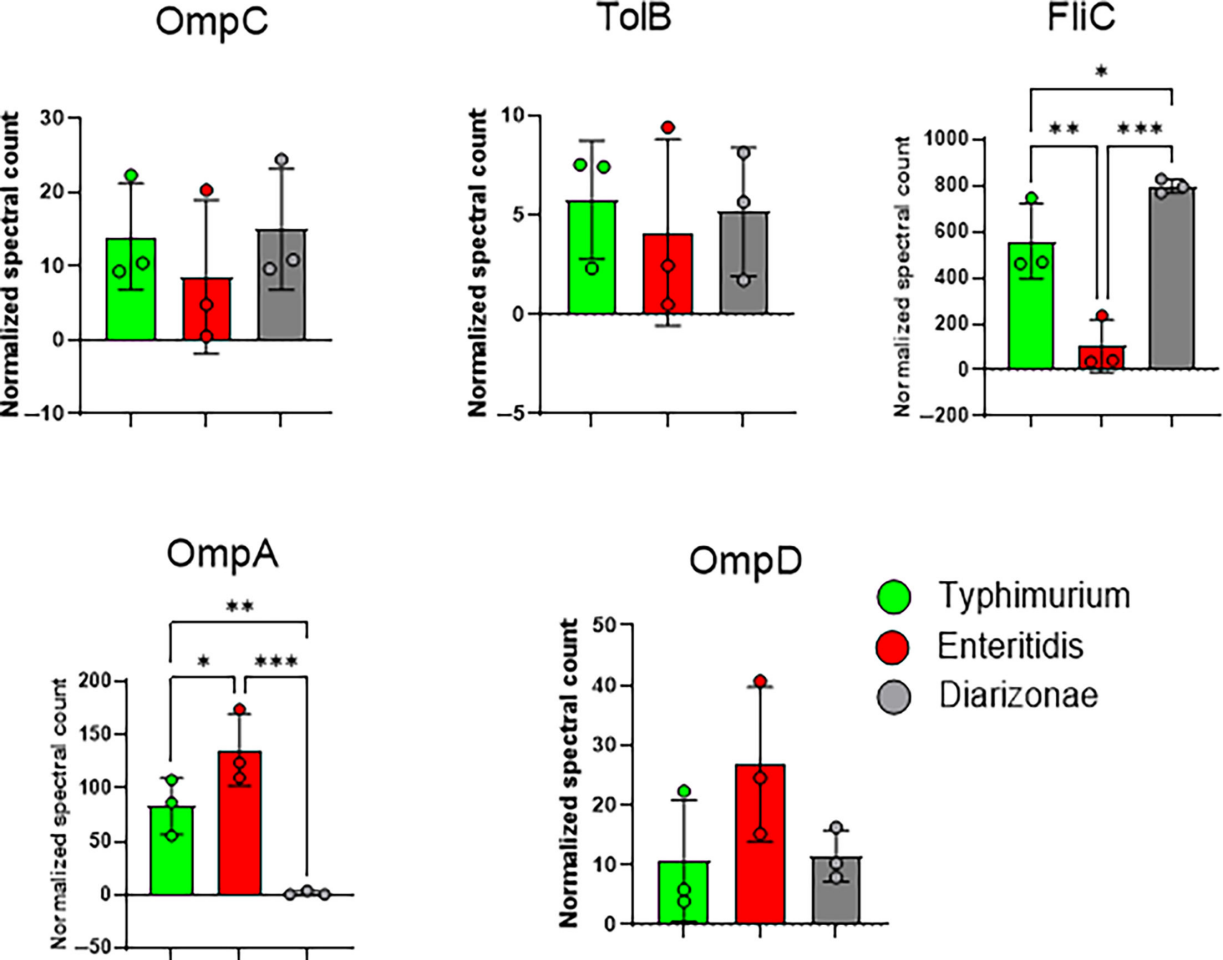
Normalized spectral count for select *Salmonella* antigens detected by mass spectrometry in the antigen preparations from *S.* Enteritidis, *S.* Diarizonae, and *S. Typhimurium.* The antigens analyzed by mass spectrometry included OmpC, TolB, FliC, OmpA, and OmpD. The antigens analyzed by mass spectrometry included OmpC, TolB, FliC, OmpA, and OmpD. A one-way ANOVA was performed to assess differences in normalized spectral counts across the three *Salmonella* strains. Statistical significance is denoted by asterisks, with *P < 0.05, **P < 0.01, and ***P < 0.001.

Although the exact mechanisms behind the SIgA and IgG antibody detection of all serovars in mice vaccinated with sEVs from *S*. Typhimurium-infected macrophages remain unclear, we observed that antigens previously detected in the sEVs from infected macrophages, including OmpA, OmpC, OmpD, TolB, and FliC, were consistently present in the antigen preparations from all analyzed strains (Fig. 6). The same antigen preparations were utilized for the ELISAs (Fig. 5). Quantitative analysis revealed that OmpA was more abundant in *S*. Enteritidis and *S*. Typhimurium compared with *S*. Diarizonae, whereas FliC was more abundant in *S*. Typhimurium and *S*. Diarizonae compared with *S*. Enteritidis (Fig. 6). These proteomic findings highlight the presence of specific antigenic proteins that may contribute to the observed immune responses and provide insight into the differential antibody recognition elicited by sEV vaccination. It is important to note, however, that these proteomic comparisons were conducted on *in vitro*-grown strains, which may not fully replicate the antigenic profile of *Salmonella* during host infection.

## DISCUSSION

Previous studies have demonstrated the efficacy of sEVs in inducing protective responses in mice, including reduced bacterial loads and protection against lethal outcomes of salmonellosis, particularly in immunodeficient BALB/c mice (6, 11). However, it remained unclear whether an sEV-based vaccine would generate immunity solely against the laboratory strain used for its development (*S*. Typhimurium) or extend to other environmental strains. In our study, we evaluated the efficacy of sEV-based vaccination against two additional *Salmonella* serovars—S. Enteritidis and *S*. Diarizonae—isolated from wastewater. Our findings demonstrate that sEVs derived from macrophages infected with a laboratory strain of *S*. Typhimurium successfully induce strong antigen-specific SIgA and IgG responses not only against the homologous lab strain but also against heterologous strains, *S*. Enteritidis and *S*. Diarizonae.

The presence of antibody responses up to 4 weeks post-vaccination aligns with the establishment of a memory response, highlighting the potential of sEVs as a preventive strategy against systemic infection with intracellular bacteria like *Salmonella* (14) Notably, the antibody responses observed also extended to the heterologous strains, *S*. Enteritidis and *S*. Diarizonae, indicating the potential of sEV vaccines to provide broad immunological coverage across diverse *Salmonella* serovars. This aspect is crucial, given the diversity of *Salmonella* strains encountered in real-world environments. A novel feature of our study is the inclusion of clinically relevant strains, such as *S. enterica* subsp. Diarizonae and serovar Enteritidis, sourced from wastewater. Unlike conventional approaches that focus primarily on limited lab strains, this approach emphasizes the need to address strains associated with human disease. By expanding to environmental strains, our findings provide a more comprehensive perspective on the protective potential of sEV-based vaccines against diverse *Salmonella* serovars.

Proteomic characterization of the three *Salmonella* strains provided insights into antigenic proteins potentially contributing to the observed immune responses. Specific proteins, including OmpA and FliC, showed differential expression among the strains, and their presence in sEVs likely played a significant role in generating the antibody responses observed. Our previous study identified these antigens in sEVs derived from S. Typhimurium-infected macrophages (11), suggesting that sEVs can effectively deliver key antigens to the immune system, potentially offering protection against diverse *Salmonella* serovars that express these proteins. However, it is important to acknowledge a limitation of this study: the proteomic data were derived from *in vitro*-grown strains, which may not fully reflect the antigenic profiles of *Salmonella* under *in vivo* conditions. Antibody responses elicited by sEVs in a host environment could be more targeted to serovars, as antigen expression may shift in response to host factors. Although our findings suggest broad immunological coverage across multiple serovars, sEV-induced antibodies may display greater specificity for antigens that are expressed during *in vivo* infection.

Moreover, the study additionally highlights the role of WBE as a tool for environmental surveillance, offering a more comprehensive and inclusive view of NTS dynamics within a population. Although clinical surveillance captures symptomatic cases, WBE’s ability to identify subclinical infections offers additional insights into NTS prevalence and circulation. This approach complements traditional methods, enhancing our understanding of infectious disease patterns. Although clinical case data from the same region and timeframe (2019–2020) were unavailable, previous studies in Florida (2017–2018) provided valuable context for NTS epidemiology (27). Analysis of laboratory-sequenced *Salmonella* isolates from infected individuals during these years identified the top five most common serotypes: Enteritidis, Newport, Javiana, Sandiego, and Braenderup (27). Notably, the serotypes detected through WBE in this study—Braenderup, Enteritidis, Javiana, and Newport—also matched prevalent clinical cases from 2 years earlier (27). This consistency underscores WBE’s potential to reflect epidemiological trends and enhance public health responses by providing a more comprehensive view of infectious disease dynamics.

Previous studies conducted in Florida (2017–2018) identified specific *Salmonella* serovars in poultry, with isolates recovered from poultry litter samples from commercial farms (28). The most frequently observed serovars in poultry litter included Typhimurium, Mbandaka, Kentucky, Enteritidis, Montevideo, Infantis, Meleagridis, Braenderup, and Muenster. Additionally, serovars Alachua, Falkensee, Liverpool, and several isolates from *S*. enterica subsp. arizonae were detected, although Diarizonae strains were not identified in these samples (28). In comparison, our current WBE study identified several overlapping serotypes, such as Typhimurium, Braenderup, Enteritidis, Javiana, and Newport, along with Diarizonae strains, which were absent in previous studies, potentially due to differences in sample year or source. The presence of serotypes like Typhimurium, Braenderup, Enteritidis, Newport, and Javiana across poultry litter (28), clinical cases (27), and wastewater highlights the importance of a One Health approach to *Salmonella* surveillance (29). Additionally, it is worth noting that certain serovars were uniquely detected by specific methods; for instance, Sandiego was uniquely identified in clinical studies (27), whereas Diarizonae was uniquely detected in our WBE analysis.

In conclusion, integrating WBE with traditional surveillance methods offers a comprehensive approach to monitoring NTS and other infectious diseases, strengthening public health responses and interventions. By incorporating WBE, our study evaluates the potential of sEV-based vaccines against circulating *Salmonella* strains, using cross-reactive antibody responses as an indicator of the vaccine’s potential for broad immunogenicity—an essential factor given the diversity within NTS. Although comparative analysis with the lab strain of *S*. Typhimurium provides a useful baseline, further research involving a wider range of clinical strains is essential to confirm the vaccine’s versatility. Moreover, to fully understand its protective potential, protection studies are needed to evaluate the vaccine’s efficacy in real-world settings.

The inclusion of WBE not only enhances pathogen surveillance but also serves as a valuable tool for testing and refining vaccine strategies. By combining WBE with traditional surveillance and building on the promising results of sEV-based vaccination, this approach establishes a robust framework for developing effective preventive measures against NTS infections. Ultimately, this integrated strategy holds significant potential for advancing public health by facilitating more targeted interventions and timely responses to emerging infectious threats.

## MATERIALS AND METHODS

### Wastewater collection and storage

Weekly composite influent wastewater samples were systematically collected from two water reclamation facilities in the City of Gainesville, FL, spanning from September 2020 to September 2021. These samples, representing a comprehensive influent mix, were aliquoted into 250 mL portions and stored at −80°C until 24 hours before the isolation process. At this point, the wastewater samples were thawed at 4°C, and 40 mL aliquots were subjected to subsequent isolation and testing (see below).

### Quantitative polymerase chain reaction

After nucleic acid extraction, weekly composite wastewater samples were subjected to quantitative polymerase chain reaction (qPCR), targeting the *invA* gene as it is specifically associated with *Salmonella* (30). Positive samples, as identified by qPCR, were advanced to the subsequent *Salmonella* isolation phase.

### *Salmonella* culture and isolation methods

*Salmonella* isolation from wastewater was achieved through an adapted version of EPA method 1682 (31). In short, TSB was inoculated with qPCR-positive wastewater and incubated for 24 hours at 37°C under various conditions: 20 mL of wastewater and 10 mL of 3X TSB; 10 mL of wastewater and 5 mL of 3X TSB; and 1 mL of wastewater and 10 mL of 1X TSB. Each condition was executed in a single replicate. After the 24-hour incubation period, six 30 µL drops from each TSB inoculation were placed on MSRV media and incubated for 17 hours at 42°C. Subsequently, spots from MSRV plates exhibiting halos were quadrant-streaked onto XLD media and incubated for 21 hours at 37°C. From each suspected positive wastewater sample, five colonies were streaked on XLD media once again, followed by incubation in Luria-Bertani (LB) for 16–19 hours at 37°C. These isolates were then preserved as glycerol stocks at −80°C.

### Sequencing

Following strain selection, the genomic characterization of *Salmonella* isolates was conducted through advanced sequencing techniques. The construction of genomic libraries was accomplished utilizing the Nextera XT DNA Library Preparation Kit. Illumina iSeq 100 System was employed for the sequencing process. The raw sequencing data underwent *de novo* assembly using the SPAdes software. This assembly strategy, which reconstructs the complete genome without the need for a reference genome, was crucial for obtaining comprehensive insights into the genomic makeup of the *Salmonella* strains under investigation.

### NTS wastewater strain selection

As part of the outlined strategy, two distinct wastewater isolates of NTS were chosen for further investigation. *S. enterica* subsp. Diarizonae (61:c:1,5,7; Table 1), selected for its absence in clinical isolates, harbors virulence factors, including the *cdtB* gene encoding cytolethal distending toxin B (32), typically associated with *S. enterica* serovar Typhi, a Typhoidal serovar. Conversely, serovar Enteritidis (9:g,m:-; Table 1) was chosen for its clinical relevance, having been identified in two separate clinical isolates during the sampling period and also being the most prevalent NTS serovar in the United States.

### Gentamicin protection assay

Prior to the gentamicin protection assay (GPA), a growth curve using the laboratory strain *S.* Typhimurium UK-1*,* and the two wastewater-isolated strains, was performed. For the GPA, 1 × 10^6^ RAW 264.7 macrophage cells were cultured in a 6-well tissue culture plate and left to attach in the 37°C incubator overnight. Overnight cultures of bacterial strains were prepared in LB broth. Cells were washed with Hank’s Balanced Salt Solution (HBSS) and left in antibiotic-free Dulbecco's Modified Eagle Media (DMEM) before being infected with *Salmonella*. The OD_600_ values of each strain from the growth curve were used to calculate the volume of bacterial culture needed to infect cells at an MOI of 30. The cells were infected with *Salmonella* for 1 hour, after which fresh DMEM containing 100 µg/mL gentamicin was added to eliminate extracellular bacteria for 1 hour. Depending on the experiment, the cells were either lysed at the 2-hour time point, or the media was replaced with DMEM containing 20 µg/mL gentamicin for an additional 24 hours. Following incubation, the macrophages were lysed with 0.1% Triton-X 100 for 15 minutes. Serial dilutions of the lysates were then plated on LB agar to assess colony counts, evaluating the invasion and survival of the different *Salmonella* strains.

### NTS sEV vaccine

The sEV vaccine, engineered following the methodology described previously (6) (Fig. S1), involved infecting RAW264.7 macrophages with *S*. Typhimurium at an MOI of 5 for 24 and 48 hours and combined. Following infection, the cell culture supernatant containing secreted sEVs was harvested and resuspended in PBS supplemented with a protease inhibitor cocktail (EDTA-free; Roche, USA). Subsequently, the suspension was filtered through a 0.22 µm polyethersulfone (PES) filter. The samples underwent a series of centrifugation steps to eliminate cellular debris and bacteria. Initially, centrifugation was performed at 500 × *g* for 10 minutes, followed by a subsequent centrifugation at 2,000 × *g* for 10 minutes, and finally, at 16,000 × *g* for 40 minutes. The resultant supernatant, devoid of cellular debris and bacteria, was carefully transferred to a fresh vial and subjected to ultracentrifugation at 100,000 × *g* for 180 minutes using an SW 32 Ti rotor and Optima XPN ultracentrifuge (Beckman, USA). Upon completion of ultracentrifugation, the supernatant was decanted, leaving behind the pellet enriched with sEVs. The sEV pellet underwent thorough washing with PBS through an additional round of centrifugation at 100,000 × *g*. Subsequently, the supernatant was decanted, and the sEV pellet was resuspended in sterile PBS supplemented with the protease inhibitor cocktail (Roche, USA). The isolated sEVs were subjected to characterization using NTA using ZetaView Quatt (Particle Matrix) to determine their concentration and hydrodynamic diameter. The instrument parameters were: sensitivity 82.0 and shutter speed. Data shown are from three different samples, each calculated as an average of five technical replicates per sample. Samples containing sEVs were diluted in PBS to achieve a concentration ranging from 1.0 × 10^8^ to 9.0 × 10^8^ particles/mL.

### Proteomic analysis of sEVs

The data from isolated sEVs at 24 and 48 hours hpi using the methodology described here were previously described (11). Here, we performed an analysis on this data set (11) to identify commonly enriched cellular components (CC) in the data set. Preranked GSEA was conducted on mean expression values from the 48 hpi data using the GSEA CLI (Broad Institute). The analysis employed GO Cellular Component gene sets from MSigDB (v2024, mouse) and was run with 5,000 permutations to ensure statistical robustness. Genes were ranked by mean expression intensity, with gene sets filtered to include sizes between 10 and 500. The classic scoring scheme was applied, using the maximum absolute value for probe collapsing. The top enriched gene sets were identified, and output files were saved for further analysis and visualization.

### Animal studies

The sEVs, isolated from cells at 24- and 48-hour post-infection time points, were then intranasally administered to mice in the amount of 40 µg sEVs/mouse. BALB/c female mice aged 6–8 weeks (Jackson Laboratory) were used for the *in vivo* experiments. Each mouse was anesthetized and administered 30 µL of PBS-suspended sEVs or PBS dropwise into the external nares using a micropipette. As a positive control, a live attenuated vaccine strain, *S*. Typhimurium UK-1 Δ*aro*A21419 (Δ*aroA*), was orally administered to additional groups of mice. The viable CFU inoculum of this vaccine was verified by serial dilution tests on agar plates. The sEV dosing regimen included three administrations spaced 2 weeks apart (at weeks 0, 2, and 4), with weekly blood and stool collections to assess serum IgG and fecal IgA titers. All procedures were conducted in accordance with institutional policies for animal health and welfare, under an approved protocol from the University of Florida’s Institutional Animal Care and Use Committee, which oversees the animal care program, facilities, and procedures in compliance with federal regulations. Animals were housed individually in HEPA-filtered barrier conditions within the University of Florida Animal Care Services facility.

### IgA and IgG ELISAs

IgA ELISAs were performed following a previously published protocol (6). Whole-cell antigens were prepared from the *Salmonella* serovars x3761 UK-1, wastewater-isolated *S*. Diarizonae, and wastewater-isolated *S*. Enteritidis through sonication. Stool samples were collected weekly from experimental animals, weighed, and processed into a 25% wt/vol fecal slurry solution (containing 0.01% sodium azide, 1% protease inhibitor in PBS), then homogenized. Immunoglobulin capture was performed on Nunc MaxiSorp flat-bottom 96-well plates coated with 2 µg of *Salmonella* antigen per well, incubated overnight. Following the blocking step and serial dilution of stool samples, the plates underwent incubation with the secondary antibody (goat anti-mouse IgA, cross adsorbed-HRP). Subsequent steps, including substrate addition and absorbance readings, were carried out using a Cytation 3 plate reader. Endpoint titers were defined as the reciprocal of the dilution giving an absorbance 0.1 U above the negative control. Analysis involved multiple Mann-Whitney U tests with Bonferroni correction for multiple comparisons. IgG ELISAs were performed as described previously (6, 11). Nunc MaxiSorp 96-well plates were coated with 2 µg NaOH-treated (LPS-cured) *Salmonella* Ag per well and incubated overnight. After the blocking step and serial dilution of sera, the plates underwent incubation with the secondary antibody (goat anti-mouse IgG, human absorbed-HRP). Subsequent steps mirrored those described for the IgA ELISA.

### Proteomic analysis of *Salmonella*

To perform proteomic analysis of *Salmonella* strains, we conducted in-gel digestion and mass spectrometry on cultures prepared as follows: stationary phase *Salmonella* cultures of *S. enterica* subsp. Diarizonae (61:c:1,5,7; Table 1), *S*. Enteritidis (9:g,m:-; Table 1), or S. Typhimurium UK-1 were centrifuged at 13,000 × *g* at 4°C, and the resulting pellets were washed with PBS containing 5 mM EDTA. Following an additional centrifugation, the pellets were washed again with PBS alone. The cells were then lysed using sonication with a Sonifier Cell Disruptor (Heat Systems-Ultrasonics), followed by centrifugation at 13,000 × *g* at 4°C to obtain the protein-containing supernatant. The supernatant was filter-sterilized using a 0.22 µm PES filter (Genesee Scientific, El Cajon, CA). For gel electrophoresis, equal amounts of protein (~50 µg per sample) from three biological replicates per strain were separated by sodium dodecyl sulfate-polyacrylamide gel electrophoresis. Each lane was excised and subjected to in-gel trypsin digestion as described previously (11, 33). The resulting peptide samples were analyzed using a 250 mm ultrahigh-performance liquid chromatography system coupled to an Orbitrap Fusion mass spectrometer (Thermo Scientific), following established protocols (11). Mass spectrometry data were analyzed using Sequest (Thermo Fisher Scientific) and X! Tandem (11). The Sequest search was performed against the FASTA *Salmonella* Uniprot database, assuming trypsin digestion, following previously published methods. Peptide and protein identifications were validated using Scaffold (Proteome Software Inc.), with peptide identifications accepted at a probability of >95.0% according to the Scaffold Local FDR algorithm. Protein probabilities were assigned using the Protein Prophet algorithm. Protein identifications were accepted at a probability of >95.0% and required at least two identified peptides. Proteins containing similar peptides that could not be differentiated based on tandem mass spectrometry (MS/MS) analysis alone were grouped, and only the top hit was reported. The reported peptide false discovery rate (FDR) was 0.35%, well below the commonly accepted threshold of 1%, and the protein FDR was 3.3%, within the typical range of ≤1%-5% for proteomics studies. Quantification was performed using normalized spectral counts, and statistical significance was assessed by *t*-test. A volcano plot was generated to visualize the differential expression of genes between the serovars. Using cutoffs of 0.176 for log10 fold change (equivalent to a fold change of ~1.5) and 1.3 for −log10 *P*-value (equivalent to a *P*-value of 0.05), genes were categorized as “Upregulated,” “Downregulated,” or “Not Significant.” The plot was created using *ggplot2* and *ggrepel* in the *R* programming environment.

## Data Availability

The mass spectrometry data are accessible through the ProteomeXchange Consortium (http://proteomecentral.proteomexchange.org) via the PRIDE partner repository with data set identifier PXD024838 (DOI: 10.6019/PXD024838). Additionally, the *Salmonella* proteome data set can be found in Ferraro, Mariola (2024), "Proteome analysis of two wastewater-isolated *Salmonella* strains," Mendeley Data, V1, DOI: 10.17632/h75yyspxv6.1.

## SUPPLEMENTAL MATERIAL

Table S1 iai.00485-24-s0001.xlsx Differential expression analysis of *S. Diarizonae* vs *S. Typhimurium* and *S. Enteritidis* vs. *S. Typhimurium*.

ASM does not own the copyrights to Supplemental Material that may be linked to, or accessed through, an article. The authors have granted ASM a non-exclusive, world-wide license to publish the Supplemental Material files. Please contact the corresponding author directly for reuse.
